# Revision Rhinoplasty in Retrocolumellar Perforation

**DOI:** 10.1007/s00266-026-05849-5

**Published:** 2026-05-04

**Authors:** Sercan Göde, Aynur Aliyeva, Mehmet Burak Apaydın, Elad Azizli

**Affiliations:** 1 Otorhinolaryngology Department, Özel Sağlık Hospital, Ege University School of Medicine, Izmir, Turkey; 2 Department of Surgery, Denver Health Medical Center, Aurora, CO USA; 3 Neuroscience Doctoral Program, Yeditepe University, Istanbul, Turkey; 4 Otorhinolaryngology Department, Ege University School of Medicine, 35100 Bornova, Izmir, Turkey; 5 Istanbul, Turkey

**Keywords:** Retrocolumellar perforation, Revision rhinoplasty, Gingivobuccal flap, Nasal tip projection, Septal reconstruction

## Abstract

**Background:**

Retrocolumellar perforations (RCPs) are rare complications of revision rhinoplasty, often associated with columellar retraction, shortened nasal length, and loss of tip projection. Their repair requires vascularized flaps that provide both mucosal coverage and structural support at the tip.

**Objective:**

To evaluate the surgical outcomes of RCP repair using endonasal and gingivobuccal flaps, with emphasis on perforation closure, nasal tip projection, and patient-reported outcomes.

**Methods:**

A cohort of 28 patients (mean age 35.6 years; 17 men, 11 women) underwent RCP repair. Most had ≥ 2 prior nasal surgeries (71.4%). Risk factors included smoking (64.3%) and cocaine use (35.7%). All patients had nasal skin contraction, short noses, and a retracted columella. Flap techniques were selected according to intraoperative anatomy, and rib cartilage grafts were used in all cases. Outcomes were assessed by perforation status, nasal tip projection, and the Standardized Cosmesis and Health Nasal Outcomes Survey (SCHNOS).

**Results:**

Perforation closure was achieved in 27 of 28 patients (96.4%). Stable and desired nasal tip projection was maintained in 23 patients (82.1%), whereas 4 patients had drooping nasal tips despite RCP closure. SCHNOS scores improved significantly from 18.2 ± 8.5 preoperatively to 89.4 ± 10.7 postoperatively (*p* < 0.001, Cohen’s d = 3.68). Among patients reconstructed with gingivobuccal flaps (n = 18), 16/18 (88.9%) maintained stable tip projection, suggesting a favorable trend but not demonstrating superiority.

**Conclusion:**

RCP repair using structured vascularized flaps achieves high closure rates, reliable nasal tip support, and significant functional and cosmetic improvement. Gingivobuccal flaps are particularly valuable in revision cases with compromised anterior support.

**Level of Evidence IV:**

This journal requires that authors assign a level of evidence to each article. For a full description of these Evidence-Based Medicine ratings, please refer to the Table of Contents or the online Instructions to Authors www.springer.com/00266.

**Supplementary Information:**

The online version contains supplementary material available at 10.1007/s00266-026-05849-5.

## Introduction

Unlike classical septal perforations, retrocolumellar perforations (RCPs) are more complex defects, frequently accompanied by columellar retraction, shortened nasal length, and underprojected nasal tips. Their actual functional and aesthetic impact depends on how successfully the reconstruction restores nasal shape, length, and projection [1–3]**.**

RCPs commonly emerge as iatrogenic complications of revision rhinoplasty**,** particularly involving rib cartilage grafts, artificial implants, or extramucosal sutures [4–7]. A typical pathophysiological sequence includes tension-induced exposure of grafted cartilage, impaired healing due to the poor granulation capability of rib cartilage, and infections—often involving Pseudomonas aeruginosa (Klebsiella and Staphylococcus*)*—which cause insidious tissue breakdown without overt inflammation. Left untreated, this process culminates in perforation and nasal tip collapse, typically manifesting within 2–6 weeks postoperatively [8–11].

Early cartilage exposure may still be managed conservatively with local antiseptics or targeted wound care to prevent perforation; however, once an RCP develops, local repair becomes ineffective, and definitive reconstruction is required. Endoscopic intranasal vascular flaps (e.g., greater palatine artery flaps) and regional skin or mucosal flaps (island, Melo, Bullhorn, vestibular mucosa) provide reliable options for closure and structural restoration. Among these, the anterior ethmoidal artery flap, greater palatine artery flap, and gingivobuccal flap are the most practical and effective choices for complex retrocolumellar perforations [12–15].

In reconstructive rhinoplasty, restoration of nasal tip projection is particularly challenging. Techniques such as the dorsal columellar strut graft, which provides stable support and precise tip positioning, have shown promising results in both primary and revision cases. Moreover, functional outcomes after rhinoplasty are increasingly assessed using validated patient-reported outcome measures, such as the Standardized Cosmesis and Health Nasal Outcomes Survey (SCHNOS), which simultaneously captures both functional and aesthetic satisfaction [16]**.**

This study aimed to describe surgical strategies for repairing RCPs and to evaluate their functional and cosmetic outcomes. Particular attention was given to the role of different flap techniques—endonasal pedicled anterior ethmoidal artery, greater palatine artery, and gingivobuccal—in achieving closure and maintaining nasal tip projection.

## Methods Section Refinement

### Study Design and Ethics

This retrospective study evaluated 28 patients who underwent closed approach revision rhinoplasty for retrocolumellar perforation RCP between January 2022 and June 2025 at Ege University Hospital and Özel Sağlık Hospital. All surgeries were performed by the same senior surgeon (SG). The study was conducted in accordance with the Declaration of Helsinki and was approved by the Ethics Committee of Ege University (Approval No: 25-5.1T/32, dated 22.05.2025). Informed consent was obtained from all participants for surgical intervention, photography, and publication of anonymized data.

### Patient Selection

Eligible patients presented with RCPs associated with columellar retraction and nasal shortening after previous rhinoplasties. Inclusion criteria were a history of revision rhinoplasty and postoperative infection that resulted in perforation. Patients with active columellar necrosis at the time of evaluation were excluded. Cases were identified from institutional records during the study period based on eligibility and documentation completeness. Patients with smoking and/or cocaine use were counseled preoperatively to stop. Compliance was assessed retrospectively using documented patient self-reports. Patients with a history of cocaine use were counseled regarding increased risk of impaired mucosal healing and advised to maintain abstinence; reconstructive planning prioritized well-vascularized flaps when healing risk factors were present.

### Outcome Measures

The primary outcome was patient-reported nasal function and cosmetic satisfaction, assessed using the 10-item SCHNOS. SCHNOS includes two subdomains: nasal obstruction (SCHNOS-O) and nasal appearance (SCHNOS-C) [16]. SCHNOS is a patient-reported measure and was completed by patients preoperatively and at follow-up; no independent rater was used. A predefined MCID threshold was not applied; interpretation was based on the magnitude of change, with statistical significance and effect size.

Scores were collected preoperatively and at six months postoperatively. Alongside patient-reported SCHNOS scores, objective outcomes were assessed, and patients were categorized as: (1) residual perforation with tip drooping, (2) closed perforation with tip drooping, or (3) closed perforation with stable (aesthetically acceptable) tip projection. Additional variables from Table 1—such as revision history, flap technique, number of perforations, associated deformities, etiology, smoking or cocaine use, and systemic diseases—were analyzed to determine their relationship with surgical success. By integrating both subjective scores (pre- and postoperative SCHNOS) and objective clinical classifications, the study aimed to capture the full spectrum of outcomes, from anatomical closure to functional and aesthetic restoration. Stable nasal tip projection was assessed clinically using standardized pre- and postoperative photographs and categorized as stable versus drooping.

**Table 1:** Patient demographics, surgical characteristics, and clinical outcomes in retrocolumellar perforation repair (N = 28)

Parameter	Subgroup		n	Percentage %
Gender	M		17	60.7
	F		11	39.3
Number of previous operations	None		2	7.1
	2 Time		8	28.6
	2< More		20	71.4
Surgical technique	Double GPA		3	10.7
	Double Buccal		6	21.4
	Right AEA, Left GPA		7	25.0
	Right AEA, Left Buccal		7	25.0
	Right GPA, Left Buccal		4	14.3
	Left AEA, Right Buccal		1	3.6
Deformity	Yes			
	Totally		5	17.9
	Columella Necrosis		4	14.3
	Short and Droopy Nose with Retracted Columella		1	3.6
	No Deformity		23	82.1
Perforation count	1		15	53.6
	2		13	46.4
Etiology	Surgery-Related Resorption (5 with cocaine use)		20	71.4
	Surgery + Infection (3 with cocaine use)		6	21.4
	Non-Surgical (both with cocaine & smoking)		2	7.1
Risk factors	Cocaine use		10	35.7
	Smoking		18	64.3
Comorbidities	Diabetes Mellitus		3	10.7
	Hashimoto’s Thyroiditis		3	10.7
Surgical Outcome Classification: Perforation Status And Nasal Tip	(1) Residual Perforation + Droopy Tip,		1	3.6
	(2) Closed Perforation + Droopy Tip,		4	14.3
	(3) Closed Perforation + Fine Nasal Tip Projection		23	82.1
*SCHNOS score and other continuous variables*				
SCHNOS score		Mid± Std	Min	Max
	Preoperative	18.2±8.5	4	33
	Postoperative	89.4±10.7	58	100
T value	7.46E-34			
*P* value	*p*<0.05			
Age (years)	35.6 ±9.3		18	52
Follow-up (months)	14±5.4		7	28

### Surgical Technique

**Fig. 1:**
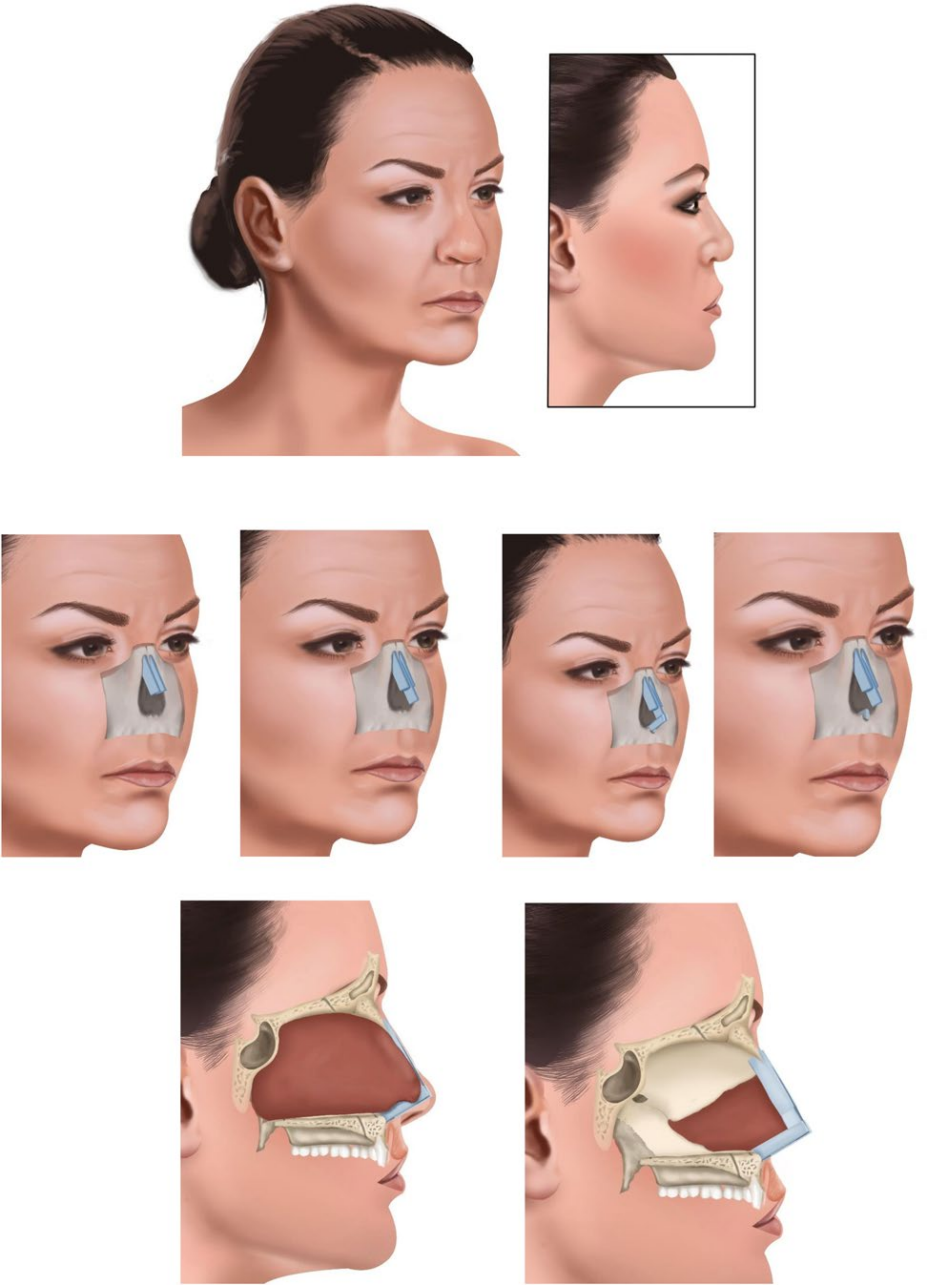
Nasal lengthening is achieved using extended spreader grafts, septal extension grafts, and double septal replacement grafts. The resulting caudal incision exposure creates a retrocolumellar defect requiring reconstruction with vascularized flaps

All patients underwent a closed rhinoplasty under general anesthesia. Endoscopic intranasal flaps were applied in all patients. Rib cartilage was used as a graft material in all procedures. To increase the length and projection of the nose, extended spreader grafts, extension grafts, and double septal replacement grafts are used. As a result of the lengthening procedure, the incision side became open, requiring reconstruction using various flap techniques (Fig. 1). Unilateral or bilateral greater palatine artery flap and anterior ethmoid artery flap were used as endonasal flaps. A unilateral or bilateral GB flap was used as a regional mucosal flap (Video).

### Anterior Ethmoidal Artery Flap

This is one of the most versatile and used endoscopic techniques. Perforations are indicated at multiple locations, each approximately 2 cm in diameter. It is a unilateral flap based on the rotation of the septal mucosa supplied by the septal branches of the anterior ethmoidal artery. Osteocartilaginous support must be present. The mean distance between the septal projection of the middle turbinate axilla and the AEA entrance in the nasal septum is 7.35mm (1).

*Posterior incision:* A vertical septal incision is made at the perpendicular plate–sphenoid rostrum junction and then extended along the choanal arch and posterior septum to the nasal floor and posteriorly to the posterior inferior meatus.

*Anterior incision* starts at the level of the middle turbinate and descends vertically through the nasal septum to the posterior edge of the perforation. It continues anteroinferiorly to the anterior limit of the inferior meatus.

*Lateral incision* joins the anterior and posterior incisions along the lateral limit of the inferior meatus without damaging the lacrimal duct valve. Once all incisions have been made, the entire mucosa of the inferior meatus, nasal floor, sphenoid rostrum, and remnant septal area is raised and rotated anteriorly until the perforation is completely covered.

The edges of the perforation are refreshed, and the flap is sutured to the remnant septal mucosa with absorbable stitches. Silicone nasal splints are fixed to the columella for 3–4 weeks. [2]

**Fig. 2:**
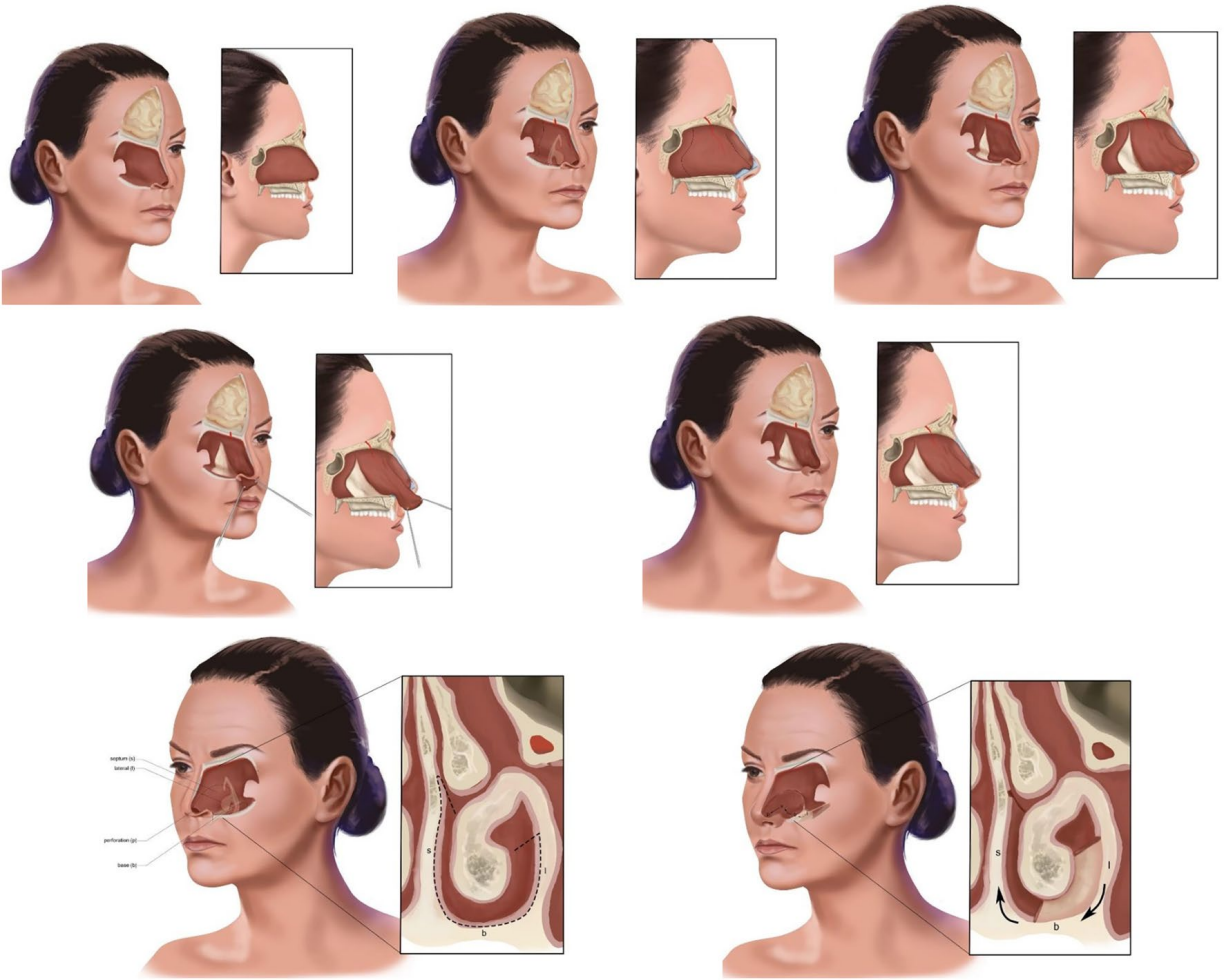
The anterior ethmoidal artery flap: a unilateral, pedicled septal mucosal flap rotated to provide robust coverage of the defect. Its extended pedicle (“long-road” pathway) allows complete closure of defects where standard local flaps are insufficient (2.1). The greater palatine artery flap: a unilateral mucoperiosteal–mucoperichondrial rotation flap pedicled through the incisive canal, providing a large, well-vascularized surface for closure of wide anterior septal defects. Its reliable blood supply and broad arc of rotation allow effective reconstruction where local septal flaps are insufficient (2.2)

Unlike routine septal perforation repairs, this technique requires a longer pedicled pathway (“long road”) and, consequently, a longer suture line, distinguishing it from standard approaches. The main clinical advantage is its ability to provide robust coverage of anteriorly located perforations, where local septal flaps may be insufficient. (Fig. 2 1 st and second line). [13, 15].

### Greater Palatine Artery (GPA) Flap

The GPA is one of the main vessels that supply the nasal septum. Interestingly, it can be located by entering the nasal septum through a relatively constant anatomical landmark. This incisive canal is located approximately 1.5cm posterior to the anterior nasal spine (ANS). This is a unilateral rotation technique that harvests a quadrangular flap of the septal and nasal floor mucosa pedicled to the GPA.

The GPA flap, pedicled through the incisive canal, was planned using CT-based pedicle localization and elevated endoscopically via vertical septal incisions connected inferiorly to permit mucoperichondrial elevation to the septum–floor junction while preserving the GPA pedicle. After margin refreshment, the flap was advanced to cover the defect and secured with absorbable sutures.

Its key strength is a reliable vascular pedicle that supplies a large mucosal surface, enabling closure of even wide anterior defects. During harvest, dissection is performed anterior to the incisive canal to avoid injuring neurovascular structures, and care is taken to protect the olfactory region. Clinically, this flap is most valuable for large anterior perforations, where it provides a well-perfused and stable lining that supports long-term closure and healing (Fig. 2 3rd line) [17, 18].

### Gingivobuccal Flap

The gingivobuccal flap (GBF) is a reliable option for reconstructing retrocolumellar perforations and anterior septal defects, particularly in revision rhinoplasty, where local septal mucosa may be insufficient. Several authors have described its use for retrocolumellar perforations, caudal septal defects, cartilage exposure, and mucosal loss following infection or implant-related complications.

**Fig. 3:**
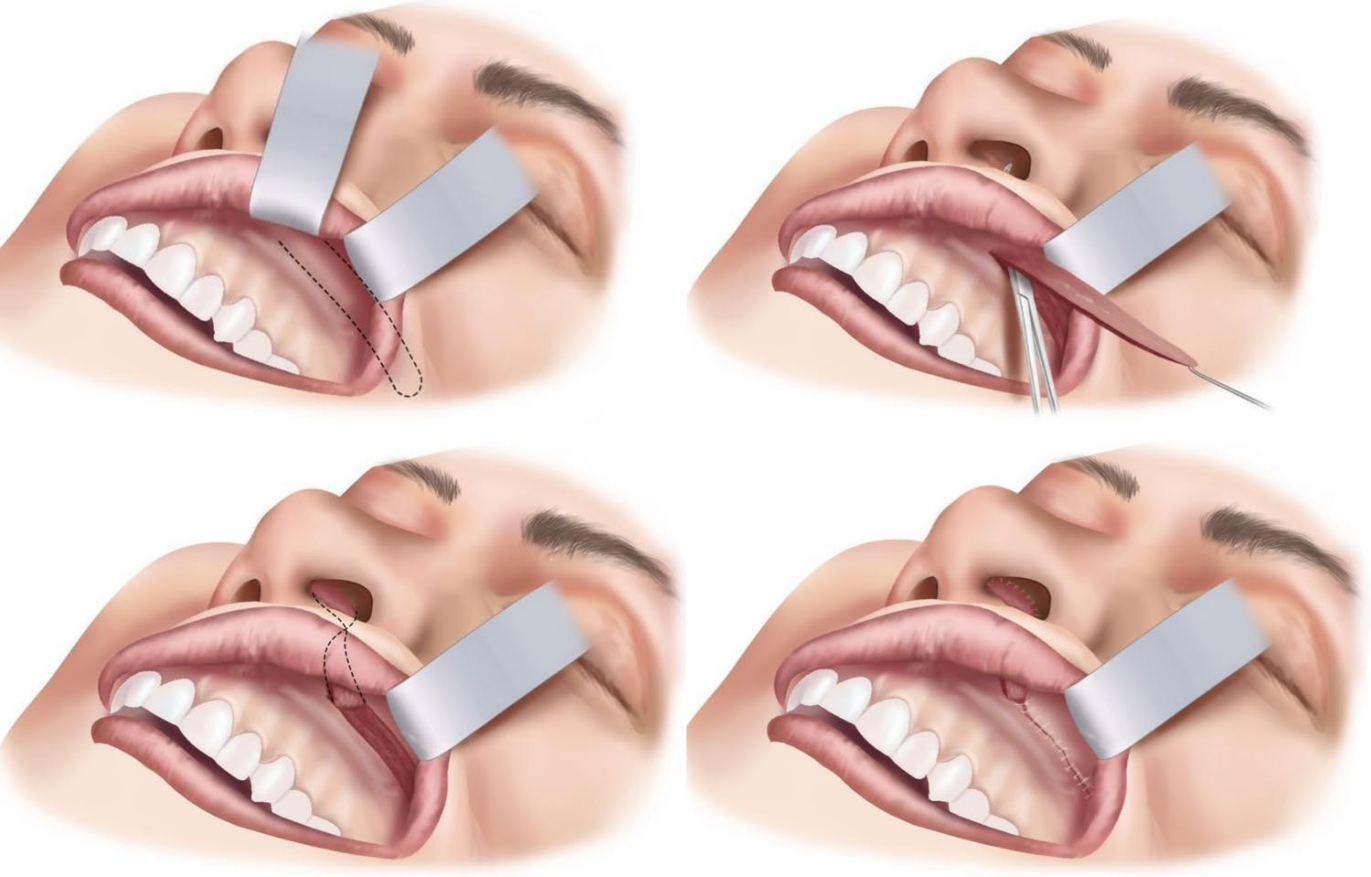
The gingivobuccal flap (GBF): an oral vestibular mucosal flap tunneled to the anterior septum to provide well-vascularized lining and structural support in retrocolumellar perforations and complex revision cases. Its robust vascular supply and ability to reinforce tip-supporting grafts make it particularly valuable when local septal mucosa is insufficient

The GBF is harvested from the oral vestibule 3–5 mm medial to Stensen’s duct, supplied primarily by superior labial artery branches, and tunneled near the anterior nasal spine to reconstruct the anterior septum and support tip-stabilizing cartilage grafts. The donor site is closed primarily with minimal morbidity. From a clinical standpoint, the gingivobuccal flap is particularly useful in complex revision cases with contracted mucosa, retrocolumellar perforation, or columella retraction. It can be used unilaterally or bilaterally, but survival rates are generally higher with unilateral harvest. The flap can also be combined with intranasal vascular flaps (AEA or GPA) to increase survival and provide an additional safety margin (Fig. 3) [19–21].

### Preoperative Considerations and Postoperative Treatment

**Fig. 4:**
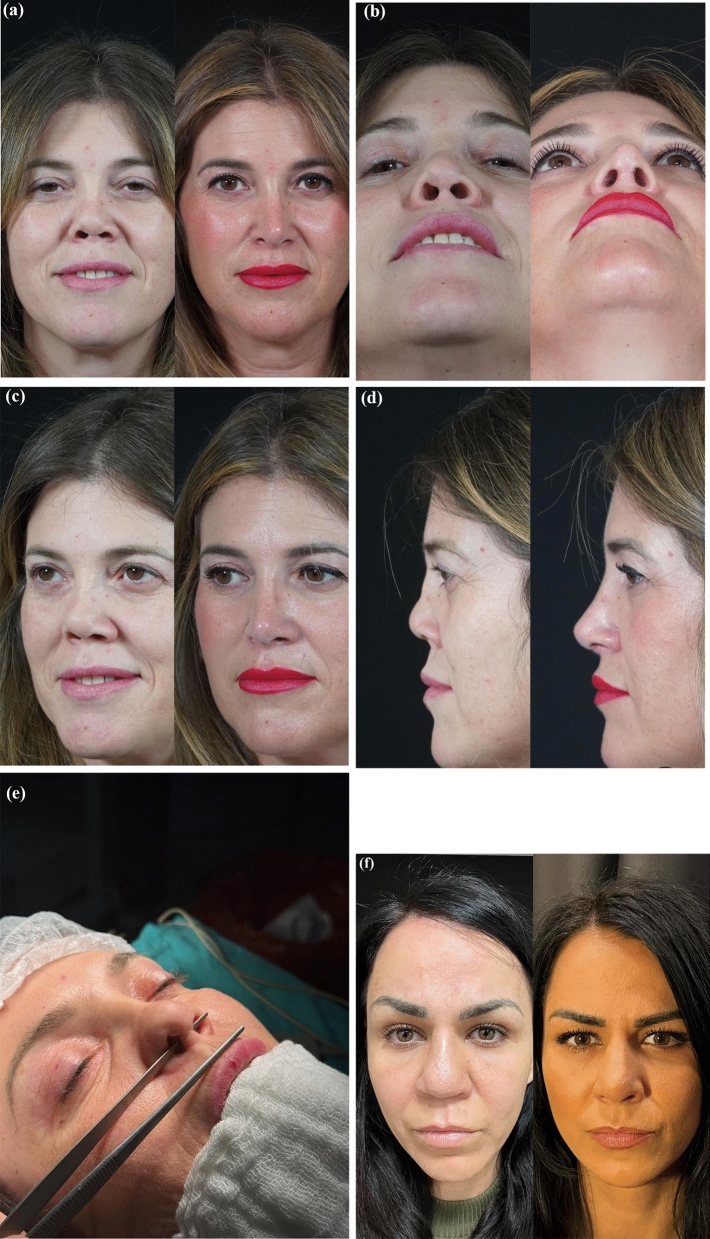

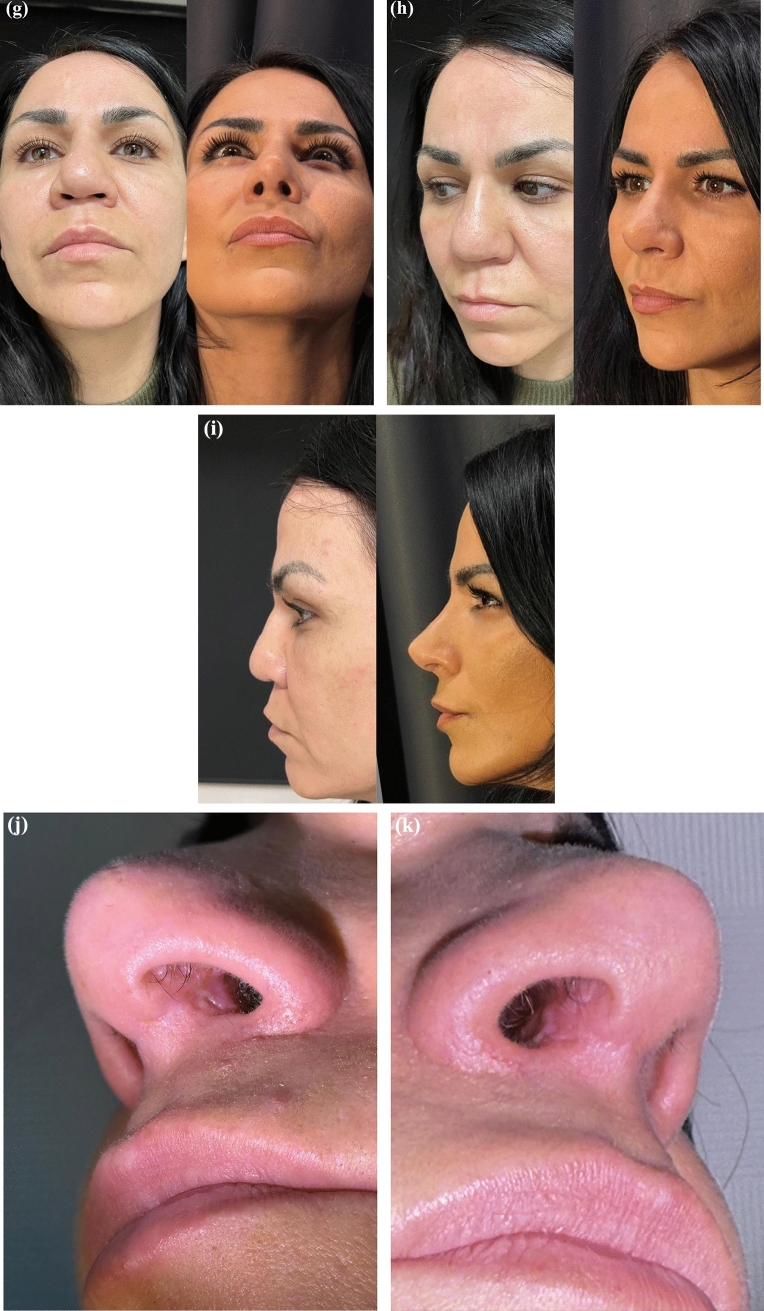
Preoperative and postoperative photographs of patients. Patient N1, **a** Front view, **b** Base view, **c** Oblique view, **d** Side view, **e** Intraoperative view of retrocolumellar perforation; Patient N2, **f** Front view, **g** Base view, **h** Oblique view, **i** Side view, **j** Long-term postoperative outcome following a left-sided gingivobuccal flap, demonstrating stable mucosal integration and sustained anterior septal reconstruction. **k** Long-term postoperative result of a right-sided anterior ethmoidal artery flap, showing durable mucosal integration and stable closure of the anterior septal defect

In all cases, antibiotic treatment was administered for 3 weeks following the procedure. Hyperbaric oxygen therapy was administered to all patients twice weekly for 1 month. Following surgery, all patients were monitored for approximately six months, and the primary outcomes assessed were RCP closure, columella length, and tip projection (Fig. 4).

### Statistical Analysis

Statistical analyses were performed using SPSS 29.0 and Python 3.11, with significance set at p < 0.05. Continuous variables were summarized as mean ± SD, and categorical variables as counts and percentages. SCHNOS distributions were checked with the Shapiro–Wilk and variance homogeneity with Levene’s test. Pre- and postoperative SCHNOS scores were compared using paired t-tests or Wilcoxon tests when assumptions were not met, and effect sizes were reported as Cohen’s d. Associations among clinical variables were evaluated with Pearson and Spearman correlations, and differences in surgical outcomes across flap types were analyzed using Chi-square and likelihood-ratio tests. This framework enabled a comprehensive assessment of both functional (SCHNOS improvement) and anatomical outcomes.

## Results

### Patient and Operative Profile

Twenty-eight patients underwent retrocolumellar perforation (RCP) repair. The mean age was 35.6 ± 9.3 years (range 18–52), and the mean follow-up was 14 ± 5.4 months. Seventeen patients were men (60.7%), and eleven were women (39.3%). Most patients (71.4%) had undergone two or more prior nasal surgeries. Perforation count was one in 53.6% and two in 46.4% of cases.

Associated deformities were present in 5 patients (17.9%), including 4 with columella necrosis and 1 with a short droopy columella. The main etiological factor was surgery-related resorption (71.4%), followed by surgery with infection (21.4%), and non-surgical causes (7.1%). Risk factors were frequent: smoking in 64.3% and cocaine use in 35.7% of patients. Comorbidities included diabetes mellitus (10.7%) and Hashimoto’s thyroiditis (10.7%). Details of demographics, risk factors, and surgical characteristics are summarized in Table 1, and full patient data are presented in Table 2.

**Table 2:** Individual patient data: demographics, surgical technique, risk factors, and outcome parameters

Age	Gender 1-Man, 2-Female	Follow-Up (Month)	Operation-Revision Number	Technique	Perforation count	Deformity	Know Course	Cocaine 1-yes 0-no	Smoking 1-yes 0-no	Vasculitis, Systemic Disease	Perforation Status And Nasal Tip Contour (1) Residual perforation + droopy tip, (2) Closed perforation + droopy tip, (3) Closed perforation + stable, aesthetic tip	Pre-SCHNOS scores	Post-SCHNOS score
44	1	8	4	Double GPA	2	All short and droopy with retracted columella	Surgery, infection,	1	1	0	3	15	85
46	2	7	9	Right AEA, left GPA	1		Surgery, resorption	0	1	0	3	33	100
50	1	10	8	Right AEA, left GPA	1		Surgery, infection	0	1	0	3	22	86
34	1	12	0	Right AEA, left buccal	2			1	1	Diabetes mellitus	3	4	90
45	1	13	2	Right AEA, left buccal	1		Surgery, resorption	0	1	0	3	12	100
32	2	9	3	Double buccal	1		Surgery, resorption	0	1	0	3	18	96
45	2	8	12	Double buccal	2	Columella necrosis	Surgery, resorption,	1	0	Hashimoto’s thyroiditis	1	28	58
47	1	12	3	Double buccal	1		Surgery, resorption	0	1	0	3	20	90
26	1	11	2	Right GPA, left buccal	2		Surgery, infection,	1	1	Diabetes mellitus	3	12	100
24	1	16	2	Right AEA, left buccal	1		Surgery, resorption	0	1	Hashimoto’s thyroiditis	3	24	92
44	2	17	10	Right AEA, left buccal	2	Columella necrosis	Surgery, resorption,	1	0	0	3	6	89
52	2	12	0	Double buccal	2			1	1	0	2	15	76
44	1	11	7	Double buccal	2	Columella necrosis	Surgery, infection	0	1	0	2	12	80
28	2	9	5	Left AEA, right buccal	1		Surgery, resorption	0	0	Hashimoto’s thyroiditis	3	10	96
28	1	8	9	Right AEA, left GPA	1		Surgery, resorption	0	1	0	3	33	92
30	1	13	8	Right AEA, left buccal	1		Surgery, resorption	0	1	0	3	28	82
25	2	14	5	Double GPA	2		Surgery, infection,	1	1	Diabetes mellitus	2	23	60
37	2	19	3	Double GPA	2		Surgery, resorption	0	1	0	3	18	96
40	2	22	3	Right AEA, left GPA	1		Surgery, resorption,	1	1	0	3	20	98
33	1	19	3	Right AEA, left GPA	2		Surgery, resorption,	1	1	0	3	28	92
41	1	17	2	Right GPA, left buccal	2	Columella necrosis	Surgery, resorption,	1	0	0	3	10	92
36	1	18	3	Double buccal	2		Surgery, infection	0	1	0	2	22	88
34	2	21	2	Right AEA, left buccal	1		Surgery, resorption	0	0	0	3	14	86
31	2	28	2	Right AEA, left buccal	1		Surgery, resorption	0	0	0	3	24	90
18	1	25	2	Right GPA, left buccal	1		Surgery, resorption	0	0	0	3	11	93
20	1	13	2	Right AEA, left GPA	2		Surgery, resorption	0	0	0	3	9	96
35	1	15	6	Right AEA, left GPA	1		Surgery, resorption	0	0	0	3	32	100
27	1	12	4	Right GPA, left buccal	1		Surgery, resorption	0	0	0	3	7	100

### Surgical Outcomes

Perforation closure was achieved in 27 of 28 patients (96.4%), with only one patient (3.6%) having a residual perforation. Stable nasal tip projection was maintained in 23 patients (82.1%). Four patients (14.3%) had closed perforations but persistent tip drooping. The single residual perforation was also accompanied by tip drooping. No significant complications, such as flap necrosis, were observed. Gingivobuccal donor-site morbidity was mild and resolved spontaneously.

### Patient-Reported Outcomes

SCHNOS scores improved significantly from a mean of 18.2 ± 8.5 preoperatively to 89.4 ± 10.7 postoperatively (*p* < 0.001), with a very large effect size (Cohen’s d = 3.68, 95% CI for mean difference: 61.9–74.8). These findings confirm that patients experienced major improvement in both nasal function and appearance (Table 3).

**Table 3:** Statistical summary of data preprocessing, outcome, and correlation analysis

Analysis component		Result
Shapiro–Wilk test	Pre-op SCHNOS	W = 0.949, p = 0.138
	Post-op SCHNOS	W = 0.682, p < 0.001
Levene's test (Variance Homogeneity)		p = 0.351
Paired t-test (SCHNOS Change)		t = 20.83, p < 0.001
Wilcoxon signed-rank test (SCHNOS change)		Statistic = 0, p < 0.001
Effect size (Cohen’s d)		d = 3.68 (Very Large)
95% confidence interval (SCHNOS change)		[61.92, 74.79]
Chi^2^ statistic		15.07
Degrees of freedom		10;p-value = 0.13
Likelihood-ratio chi-square		Statistic: 15.32; p-value: 0.121
Pearson		
Age vs revision number		r = 0.40
Revision number vs pre-op SCHNOS		r = 0.56
Age vs post-op SCHNOS		r = 0.36
Pre-op vs post-op SCHNOS		r = 0.18
Revision count vs postop SCHNOS		0.01
Perforation count vs postop SCHNOS		0.11
Spearman		
Age vs revision number		ρ = 0.32
Revision number vs pre-op SCHNOS		ρ = 0.49
Age vs post-op SCHNOS		ρ = 0.03
Pre-op vs post-op SCHNOS		ρ = 0.12

### Risk Factors and Correlations

**Fig. 5:**
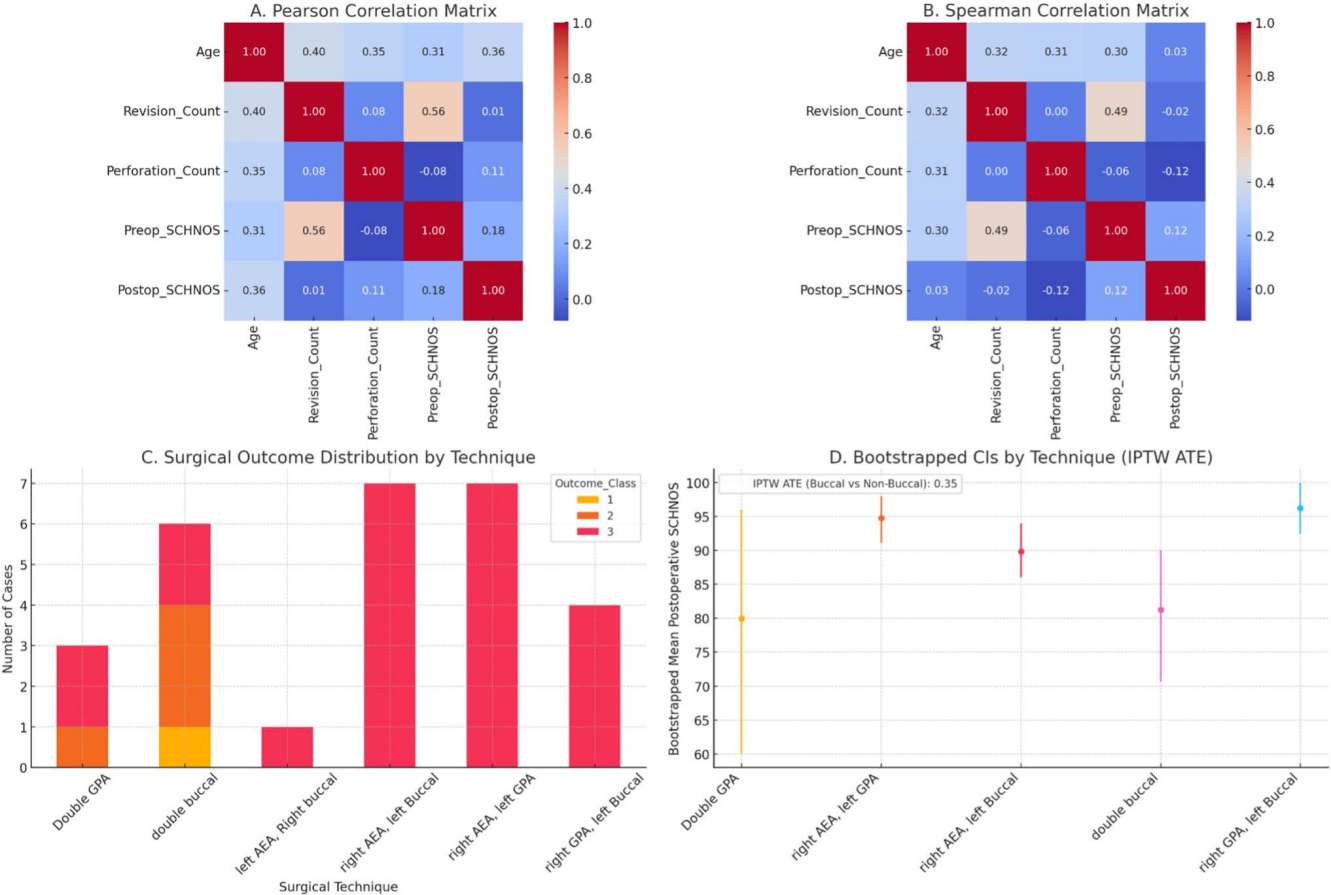
Correlation matrices of age, revision history, perforation count, SCHNOS scores, and surgical technique effectiveness, **A** Pearson correlation matrix, **B** Spearman correlation matrix, **C** Surgical outcome distribution by technique, **D** Bootstrapped CIs by surgical technique *Note*: Surgical techniques demonstrated varying bootstrapped mean postoperative SCHNOS scores, while IPTW analysis revealed a modest treatment effect (ATE = {≈ value}) favoring buccal-based flaps over others for closure and tip stabilization outcomes

Correlation analyses (Table 3, Fig. 5A–B) showed that revision number correlated with preoperative SCHNOS (r = 0.56, ρ = 0.49), age correlated moderately with revision number (r = 0.40), and postoperative SCHNOS had only weak or negligible correlations with age, revision count, or perforation count. From a clinical perspective, this means that baseline patient factors did not strongly limit postoperative improvement. Residual perforation and postoperative tip drooping were predominantly observed in patients with behavioral risk factors such as smoking or cocaine use, while diabetes and thyroid disease did not prevent closure but were associated with suboptimal tip support in a minority of cases. In subgroup analyses, stable tip projection (Outcome 3, Table 2) was lower in smokers (14/18, 77.8%) and cocaine users (7/10, 70.0%) than in non-smokers (9/10, 90.0%) and non-users (16/18, 88.9%), while closure remained high across groups (≥ 90%).

### Surgical Techniques and Clinical Implications

When comparing flap types, there was no statistically significant difference in closure rates (χ^2^=15.07, *p* = 0.13). However, stable tip projection was observed more frequently in cases with a gingivobuccal flap than in those without it, which should be interpreted as a trend given the sample size and heterogeneity. Figure 5C–D shows a clear trend: Among patients reconstructed with gingivobuccal flaps (n = 18), 16 (88.9%) maintained stable tip projection and only 2 (11.1%) showed drooping despite closure. Also, in those reconstructed without gingivobuccal flaps (n = 10), only 7 (70.0%) maintained stable projection, while 3 (30.0%) had drooping or residual perforation.

## Discussion

This study emphasizes that retrocolumellar perforation (RCP) should be regarded as a distinct clinical problem, rather than a simple variant of septal perforation. RCP is often accompanied by columellar retraction, nasal shortening, and loss of tip projection, which together create both functional and cosmetic challenges. Successful management requires a reconstructive approach that restores mucosal lining, reinforces nasal tip support, and considers patient-related risk factors such as prior surgery, smoking, and cocaine use.

Despite a variety of surgical techniques, nasal septal perforation repair remains a challenging procedure, with no universally accepted standard. Approaches such as endoscopic, external, sublabial, or midfacial degloving, using diverse grafts and flaps, have shown mixed success [22]. Cassano et al. reported a 76% closure rate with a unilateral endoscopic flap, yet such methods often fall short in addressing the structural and aesthetic demands of retrocolumellar perforations. Our study, achieving an 82.1% closure rate and >70-point improvement in SCHNOS, offers a reproducible solution tailored to the unique complexity of these rare 3D defects [2].

Patients with more severe preoperative symptoms experienced the greatest postoperative improvement, with baseline SCHNOS and age emerging as the strongest predictors. Although comorbidities like diabetes and thyroid dysfunction did not reach statistical significance, prior studies by Müller et al. suggest impaired nasal mucosal healing in diabetic patients [23]. Smoking and cocaine use, known to impair vascular integrity and tissue repair, as reported by Blaise et al. [24], were also associated with poorer outcomes in our clustering analysis—echoing Tumlin et al., who found significantly higher failure rates in patients with these risk factors [25]. Although not statistically significant, diabetes, thyroid disease, smoking, and cocaine use were associated with less improvement. Cluster patterns suggested a subgroup enriched for smoking/cocaine use with higher residual perforation risk, which may help guide postoperative monitoring [1–4, 6, 7].

Unlike classical septal perforations, retrocolumellar perforations (RCPs) represent a rare, anatomical defect characterized by columellar retraction, nasal shortening, and loss of tip support—features that necessitate a fundamentally different reconstructive approach. While techniques described by Topkara [26] for 3D columellar strut support, Cassano et al. [2] for endoscopic septal perforation repair, and Hanci et al. introduced tricellular matrices for septal repair, these techniques do not fully address the aesthetic demands of RCPs. Our study presents an anatomically tailored algorithm that uniquely targets both the functional and structural challenges of this uncommon entity.

The integration of endonasal pediculated arterial flaps with GB flaps formed the foundation of our hybrid reconstructive strategy. Inspired by the robust vascularization and minimal donör-site morbidity reported by Arana-Fernández et al. [18]**,** the GPA flap provided reliable perfusion for tension-free closure, even in scarred or ischemic nasal beds. Complementing this, Squaquara R. et al. [27] emphasized the high success rate of epithelialization with GB flaps, though it is limited in larger defects. Our approach merges the strengths of both, paralleling the dual-layered success in retrocolumellar perforations. This synergy proved critical in achieving durable closure and enhanced nasal tip projection in anatomically compromised patients.

There were no significant complications such as flap necrosis, and donor-site morbidity remained minimal. Anatomical closure was achieved in 82.1% of cases, with 78.6% of patients maintaining stable tip projection. These outcomes are consistent with the report by Tumlin et al., who achieved a 73.2% closure rate using the anterior ethmoidal artery flap for septal perforation. However, they noted higher failure rates in patients with comorbidities such as diabetes and cocaine use [25]. Bergel et al. reported that columellar reconstruction using paranasal or nasolabial flaps, or composite grafts, can provide satisfactory aesthetic and functional outcomes when vascularity is preserved [1]. Compared to these approaches, our technique demonstrates favorable safety and anatomical success, even in revision cases with compromised vascularity. In our study, flap choice was treated as an algorithmic, anatomy-driven decision: Endonasal vascular flaps (AEA/GPA) were selected for reliable lining based on pedicle availability and defect extent, with gingivobuccal flaps added when anterior mucosal replacement and/or tip-support augmentation was required.

Established retrocolumellar perforations require a well-vascularized flap tailored to defect characteristics and pedicle viability. If the AEA flap is not feasible, a GPA flap can cover larger defects, while gingivobuccal flaps can add mucosal replacement and tip support; complex cases may require combined flaps and/or cartilage grafting.

This study has several strengths. It presents one of the largest reported series of retrocolumellar perforation (RCP) repairs, combining anterior ethmoidal, greater palatine, and gingivobuccal flaps in revision rhinoplasty. Reporting both anatomical closure and patient-reported outcomes (SCHNOS) provides a comprehensive view of success from both the surgeon's and the patient's perspectives.

This study is limited by its retrospective design, which is inherently subject to selection bias, incomplete documentation, and the absence of a randomized comparison. The absence of a control or comparator group limits causal inference and precludes definitive conclusions regarding the superiority of any specific flap technique. The duration of abstinence from cocaine prior to surgery could not be reliably quantified because it was inconsistently documented. Its main limitations involve heterogeneity in flap selection, the influence of high-risk behaviors such as smoking and cocaine use, and small subgroup sizes that reduce statistical power. Future studies with larger cohorts and standardized surgical protocols would allow clearer comparisons between flap types and better evaluation of comorbidities and risk factors.

## Conclusion

Retrocolumellar perforation repair using a structured approach with vascularized flaps achieved a high closure rate and significant improvement in both nasal function and appearance. Stable nasal tip projection was maintained in most patients. Complications were minimal, and outcomes were favorable even in complex revision cases. These findings support the use of gingivobuccal flaps as a useful adjunct to endonasal vascular flaps for anterior support in selected revision cases.

## Supplementary Information

Below is the link to the electronic supplementary material.

Supplementary file1 (MP4 134795 kb)

## Abbreviations

AEA: Anterior ethmoidal artery
GBF: Gingivobuccal flap
GPA: Greater palatine artery
RCP: Retrocolumellar perforation
SCHNOS: Standardized cosmesis and health nasal outcomes survey
SCHNOS-C: SCHNOS cosmesis domain
SCHNOS-O: SCHNOS obstruction domain
SG: Senior surgeon (same operator for all surgeries)
